# Host Gut-Derived Probiotic, *Exiguobacterium acetylicum* G1-33, Improves Growth, Immunity, and Resistance to *Vibrio harveyi* in Hybrid Grouper (*Epinephelus fuscoguttatus* ♀ × *Epinephelus lanceolatus* ♂)

**DOI:** 10.3390/microorganisms12081688

**Published:** 2024-08-16

**Authors:** Mingqing Zhang, Yuwei Feng, Zhongxuan Zhong, Qianping Du, Wei Yu, Jinhui Wu, Xiaolin Huang, Zhong Huang, Guangting Xie, Hu Shu

**Affiliations:** 1 School of Life Sciences, Guangzhou University, Guangzhou 510006, China; zhangmq58@mail2.sysu.edu.cn (M.Z.); fengyuwei99@163.com (Y.F.); 2014400066@e.gzhu.edu.cn (Z.Z.); 2014100022@e.gzhu.edu.cn (Q.D.); 2112114036@e.gzhu.edu.cn (G.X.); 2 School of Life Sciences, Sun Yat-Sen University, Guangzhou 510275, China; 3 Shenzhen Base of South China Sea Fisheries Research Institute, Chinese Academy of Fishery Sciences, Shenzhen 518121, China; weiyu88888888@126.com (W.Y.); huangxiaolin@scsfri.ac.cn (X.H.); huangzhong@scsfri.ac.cn (Z.H.); 4 Sanya Tropical Fisheries Research Institute, Sanya 572018, China; 5 Agro-Tech Extension Center of Guangdong Province, Guangzhou 510500, China; wjhin@sina.com

**Keywords:** hybrid grouper, *Exiguobacterium acetylicum*, growth, immunity, mariculture

## Abstract

Several exogenous probiotics are applicable in fish culture; however, challenges in isolation and verification have hindered the full utilization of numerous host probiotics. Therefore, this study aimed to apply the host probiotic *Exiguobacterium acetylicum* G1-33 to hybrid grouper (*Epinephelus fuscoguttatus* ♀ × *Epinephelus lanceolatus* ♂) cultures and explore its mechanism of action. In total, 360 hybrid grouper were divided into four groups, which were fed the following for 60 days: three received commercial feed with varying concentrations of *E. acetylicum* G1-33 (10^6^, 10^8^, and 10^10^ CFU/g), while a control group received commercial feed. The results showed that supplementation with 10^6^ and 10^8^ CFU/g of *E. acetylicum* G1-33 enhanced gut morphology, upregulated growth-related genes (*ghr1*, *igf-2*, *s6k1*, *tor*), and promoted growth, with supplementation with 10^8^ CFU/g resulting in the most notable enhancement. However, supplementation with 10^10^ CFU/g inhibited growth, possibly because of changes in intestinal morphology. Additionally, supplementation with *E. acetylicum* G1-33 upregulated the expression of immune-related genes (*c3*, *myd88*, *Cu/Zn-sod*, *tlr3*, and *tnf2*) in the liver and head kidney but led to an increase in malondialdehyde content, as well as a decrease in alkaline phosphatase and acid phosphatase activities, in the liver and serum, indicating increased oxidative stress. Moreover, supplementation with 10^6^ and 10^8^ CFU/g *E. acetylicum* G1-33 enhanced the widespread expression of immune-related genes in the head kidney and liver, respectively, and improved resistance to *Vibrio harveyi*, whereas supplementation with 10^10^ CFU/g weakened this resistance. In conclusion, *E. acetylicum* G1-33, particularly at 10^8^ CFU/g, emerged as an effective probiotic, optimizing growth performance and immunity in hybrid grouper. This research is pioneering in its application of *E. acetylicum* in mariculture, potentially broadening the range of probiotic strategies in aquaculture.

## 1. Introduction

Aquaculture, which refers to the controlled culture of aquatic organisms, has emerged as a pivotal sector in meeting the increasing global demand for aquatic products [1]. However, the intensification and expansion of aquaculture also present a series of challenges, including disease outbreaks and environmental sustainability issues [2]. Recently, the application of probiotics in aquaculture has emerged as a promising strategy for addressing these challenges and enhancing the productivity and sustainability of this industry [3,4]. Probiotics are living microorganisms widely acknowledged for their positive effects on human and animal health when consumed in moderation [5]. Currently, the use of probiotics in aquaculture can enhance the growth, disease resistance, and overall performance of cultured species while reducing the reliance on antibiotics and other chemicals [6,7]. For example, Abdel-Latif et al. [3] confirmed that probiotic supplementation could promote the growth of *Pangasianodon hypophthalmus* and enhance its resistance to *Aeromonas hydrophila* by boosting immunity. Similar results have been reported for *Oreochromis niloticus*, *Litopenaeus vannamei*, *Labeo rohita*, and *Oncorhynchus mykiss* and a variety of probiotics, including *Lactobacillus plantarum*, *Bacillus* spp., *Enterococcus faecium*, *Saccharomyces cerevisiae*, and *Latilactobacillus sakei* [4,8,9]. Moreover, probiotics can contribute to enhancing water quality by promoting nutrient cycling, reducing organic waste, and mitigating harmful algal blooms, thus promoting the welfare of cultured species and improving the stability and resilience of aquaculture systems [10,11].

Mariculture is an important component of the aquaculture industry and plays a crucial role in alleviating pressure on marine fishing and enhancing food security. Mariculture, which encompasses finfish, shellfish, shrimp, and algae, can provide diverse and sustainable seafood [12]. In coastal areas, mariculture is a key driver in promoting sustainable fisheries and socioeconomic development [1]. *Epinephelus* spp. have a delicate flavor, high nutritional and economic value, and high prevalence in mariculture [13]. According to the Food and Agriculture Organization (FAO) of the United Nations, global grouper production increased from 269,179 tons in 2012 to 578,776 tons in 2017, with a value of 2352.44 million USD [14]. The hybrid grouper (*Epinephelus fuscoguttatus* ♀ × *Epinephelus lanceolatus* ♂) is a new grouper breed with an improved growth rate and disease resistance, combining the advantages of its parents. Hybrid grouper have emerged as a major mariculture species, contributing to enhanced yield and profitability within the grouper culture industry [15]. However, disease outbreaks continue to hinder the development of this industry [16]. Common diseases affecting hybrid grouper include viral nerve necrosis, iridoviruses, bacteria (e.g., *Vibrio*), and parasites, which lead to high mortality rates, compromised growth performance, and overall health deterioration [17,18]. Given the successful application of probiotics in various aquaculture species, the development of probiotics specifically tailored to hybrid grouper has become crucial to ensuring sustained growth in the grouper culture industry.

Multiple studies have indicated that the gastrointestinal tract of fish harbors a rich diversity of potential probiotics with the ability to inhibit pathogen growth and successfully colonize the gastrointestinal tract [19]. In this study, *Exiguobacterium acetylicum* G1-33 was isolated from the gastrointestinal tract of hybrid grouper, and the effects of *E. acetylicum* G1-33 on the growth, immunity, and disease resistance of hybrid grouper were assessed through dietary supplementation. Jinendiran et al. [20] reported the effective inhibition of the fish pathogens *Vibrio* spp. and *A. hydrophila* by *E. acetylicum* in vitro, along with its susceptibility to broad-spectrum antibiotics. Xie et al. [21] confirmed through genome sequencing and in vitro experiments that *E. acetylicum* G1-33 is sensitive to a variety of antibiotics and has potential abilities such as epithelial adhesion, antioxidant and antibacterial activities, and essential amino acid synthesis. However, its application in marine fish remains unexplored. Thus, this study aimed to provide novel insights into the application of *E. acetylicum* G1-33, thereby diversifying the range of probiotics used in hybrid grouper cultures.

## 2. Materials and Methods

Details are provided in the Supplementary Information.

### 2.1. Ethics Statement

All experimental protocols were authorized by the Special Committee on Scientific Ethics of Guangzhou University (No. SCSEGU22062107), and adherence to the ethical standards and biosafety protocols was ensured.

### 2.2. Evaluation of Probiotic Application Potential

Experimental *E. acetylicum* G1-33 was isolated from the guts of hybrid grouper and stored in an ultralow-temperature refrigerator at the Microbiology Laboratory of Guangzhou University (Figure S1). During the experiment, the reserve strains were inoculated into Luria–Bertani broth (Sangon, Shanghai, China) at 37 °C for 24 h before being used.

The effects of differences in temperature (25, 35, and 45 °C), pH (3.5, 5.5., 7.6, and 9.5), salinity (0, 10, 20, 30, 40, and 50 ppt), and bile salt concentration (0, 0.03, 0.3, and 3% (*w*/*v*)) on the growth of *E. acetylicum* G1-33 were tested, as previously described [22]. For all tests, except the temperature tests, *E. acetylicum* G1-33 was incubated at 35 °C for 39 h in a shaker (Yiheng, Beijing, China). An ultraviolet–visible spectrophotometer (Shanghai Youke Instrument Co., Ltd., Shanghai, China) was used to determine the growth of *E. acetylicum* G1-33 under each test condition at 0, 1, 3, 5, 7, 11, 15, 23, 31, and 39 h.

After incubating *E. acetylicum* G1-33 at different temperatures, pH levels, salinities, and bile salt concentrations for 24 h, the bacterial solution was collected and centrifuged (2000 rpm, 4 °C, 20 min), and the supernatant was collected. The activities of amylase, protease, and lipase in the supernatant were determined according to the instructions of the Nanjing Jiancheng Bioengineering Institute (Nanjing, China).

### 2.3. Diet Preparation and Feeding Program

The basal diet was a commercial feed (Yuequn, Jieyang, China) with the composition shown in Table S1. Diets containing *E. acetylicum* G1-33 were prepared according to the method of Liu et al. [23], with slight modifications. In short, the isolated strains were inoculated into TSB (Huankai Guangdong, Guangzhou, China) and then incubated at 37 °C for 24 h. The bacterial cultures were then centrifuged at 3000× *g* at 4 °C for 15 min. The bacterial sediment was washed three times with sterile phosphate-buffered saline (China Shanhaisanggong) and re-suspended in PBS. *E. acetylicum* G1-33 was added to the commercial feed under sterile conditions at concentrations of 0, 10^6^, 10^8^, or 10^10^ colony forming units (CFU)/g, dried at 20 °C for 24 h, and stored at 4 °C until use. Specifically, 300 g pellet feed was packed into a 1 L conical bottle, G1-33 suspension was poured into the center of the feed, the bottle was immediately manually shaken horizontally so that the bacterial solution was evenly absorbed by the feed particles, and finally, the feed was spread on a clean plastic board, dried at 20 °C for 24 h, and stored at 4 °C for use. The viability of probiotic cells in the probiotic feed was measured by plate counting. The 0 concentration did not contain the bacterial suspension but contained the same volume of PBS. The experimental feed was prepared every 3 days.

The hybrid grouper were purchased from the Guangdong Marine Fishery Experimental Center (Huizhou, China). Hybrid grouper were disinfected and raised in the Shenzhen Base of the Chinese Academy of Fishery Sciences for 10 d, during which time they were fed commercial feed. Afterwards, 360 healthy hybrid grouper (29.75 ± 0.82 g) without body damage or parasites were selected and randomly assigned to 12 breeding buckets (170 L), with 30 fish per bucket, equipped with a water quality detection system (Haiyan Aquaculture Technology Co., Ltd., Huizhou, China). The experiment was divided into four groups that were fed *E. acetylicum* G1-33 at different concentrations, a control (C; 0 CFU/g), low-dose (L; 10^6^ CFU/g), medium-dose (M; 10^8^ CFU/g), and high-dose (H; 10^10^ CFU/g) group, with three replicates per group. The hybrid grouper were fed twice per day (07:00 and 18:00) with the diets to satiation for 60 days.

### 2.4. Growth Performance and Survival Rate

After 60 days, the number of hybrid grouper in each bucket was calculated and recorded after anesthesia with 100 mg/L tricaine methanesulfonate. After measuring weight and body length, growth performance was calculated using the following formulae:

Weight gain (WG, g) = final body weight − initial body weight

Weight gain rate (WGR, %) = 100 × [(final body weight − initial body weight)/initial body weight]

Specific growth rate (SGR, %) = 100 × [ln (final body weight) − ln (initial body weight)]/experimental days

Condition factor (CF, %) = 100 × (final body weight)/(final body length)

Visceral index (VI, %) = 100 × (visceral weight/body weight)

Survival rate (SR, %) = 100 × (final number of fish/initial number of fish)

### 2.5. Sample Collection

Three fish were randomly selected from each bucket and anesthetized using 250 mg/L eugenol (Sangon, China). Blood was collected through the tail vein and placed in 1.5 mL centrifuge tubes at room temperature for 3 h. Serum was collected by centrifugation (4 °C and 8000 rpm for 15 min), frozen in liquid nitrogen, and stored at −80 °C for further analysis.

The abdominal cavity of each fish was opened with sterile anatomical tools; the liver, muscle, spleen, and head kidney were separated; and excess attached tissue was removed. Tissue samples were transferred into sterile centrifuge tubes, rapidly frozen using liquid nitrogen, and stored at −80 °C until subsequent analysis. Finally, gut, liver, head kidney, and spleen tissues were placed in sterile centrifuge tubes containing 4% paraformaldehyde for histological examination.

### 2.6. Gene Expression Detection

Total RNA was extracted from the spleen, head kidney, liver, and muscles using TRIZOL (Vazyme, Nanjing, China). The concentration, purity, and integrity of the total RNA were determined using an ultra-microplate spectrophotometer and agarose gel electrophoresis. Complementary DNA (cDNA) was synthesized using HiScriptII RT SuperMix for qPCR (+gDNA wiper) (Vazyme, China) and stored at −80 °C until further analysis.

Growth- and immune-related gene expression in hybrid grouper was detected by real-time quantitative polymerase chain reaction (qPCR) following the method described in our previous study [24] with a LightCycler^®^ 480 Instrument II (Roche, Basel, Switzerland). *β-actin* was selected as the reference gene, and relative gene expression was calculated by the 2^−ΔΔCt^ method. The qPCR primers used for detection are listed in Table S2.

### 2.7. Biochemical Assays

Acid phosphatase (ACP), alkaline phosphatase (AKP), and malondialdehyde (MDA) levels in the liver and serum were measured using content determination kits (Nanjing Jianjieng Biotechnology Co., Ltd., Nanjing, China) according to the manufacturer’s instructions.

### 2.8. Histological Examinations

Following the method described by El-Kady et al. [25], with some modifications, gut samples were completely fixed in 4% paraformaldehyde solution and then dehydrated with a series of ethanol solutions of varying concentrations, followed by xylene soaking and paraffin embedding. Four sections (5 μm thick) were cut from each sample and stained with hematoxylin and eosin. Images of the sections were captured using a Nikon Eclipse 80i microscope (Nikon, Tokyo, Japan) equipped with a Nikon digital scope SD-MS camera and Nikon NIS-Elements 4.0 software. The image analysis software NDP view 2 was used to measure the villus width, villus height, microvillus height, submucosal layer thickness, and muscle layer thickness of the gut.

### 2.9. Challenge Test

Referring to the method described by Ren et al. [26], 10 fish were randomly selected from each bucket after 60 days of culture, and 0.2 mL of *Vibrio harveyi* (1 × 10^8^ CFU/mL, provided by the South China Sea Fisheries Research Institute, Chinese Academy of Fishery Sciences) was injected intraperitoneally. This is bacterium that causes severe diseases in maricultured fish, including grouper [14]. The grouper were fasted during the challenge test. The fish were monitored for 96 h after the challenge, and mortality rates were recorded. In addition, the livers and head kidneys were collected 48 and 96 h after challenge to detect the expression levels of immune-related genes, as described in Section 2.6.

### 2.10. Data Analysis

In this study, data were expressed as mean ± standard deviation (SD). The Levene test was used to evaluate the homogeneity of variance. Statistical differences between the data were determined by one-way analysis of variance using Tukey’s method. All statistical analyses were performed using SPSS 24 software. Statistical significance was established at *p* < 0.05. Origin 2018 software was used for all drawings.

## 3. Results

### 3.1. Application Potential of E. acetylicum G1-33

**Figure 1:**
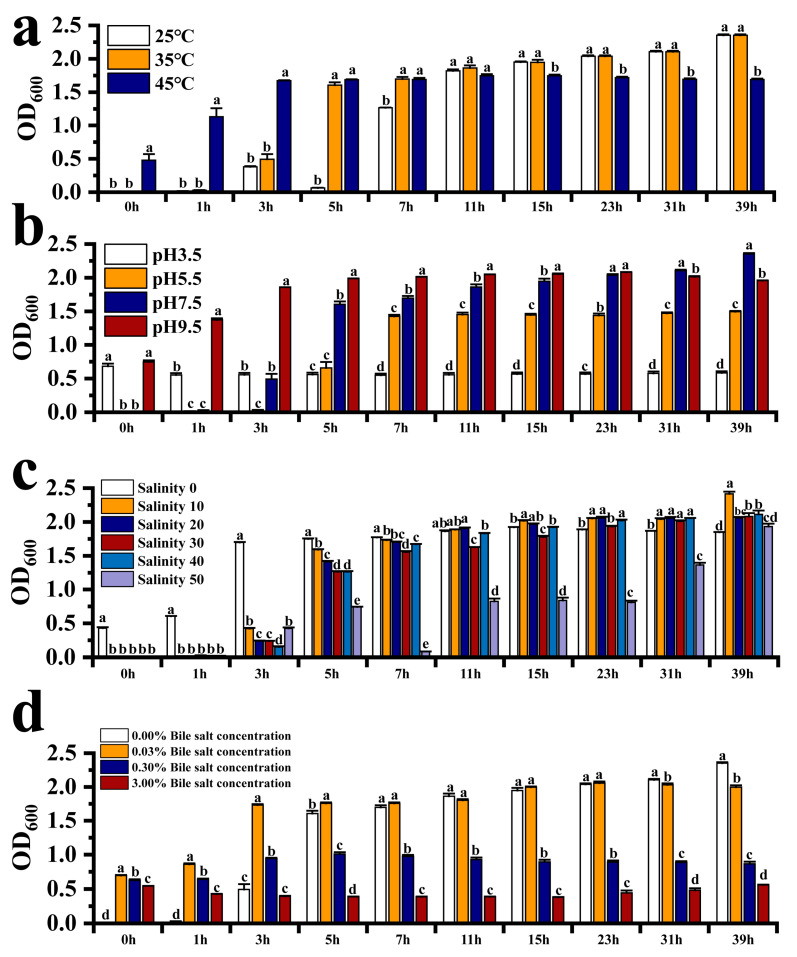
Growth characteristics of *E. acetylicum* G1-33 under different temperature (**a**), pH (**b**), salinity (**c**), and bile salt (**d**) conditions. Each value represents mean ± SD (n = 3). Bars with different superscripts significantly differ (*p* < 0.05, by one-way ANOVA).

We evaluated the potential application of *E. acetylicum* G1-33 in hybrid grouper cultures by detecting the growth and enzyme-producing activities of this strain under different conditions. The results showed that *E. acetylicum* G1-33 could grow at 25–45 °C and demonstrated higher amylase and lipase activities at 25–35 °C, whereas proteinase activity was the highest at 45 °C (Figure 1a and Figure S2). The pH test revealed that *E. acetylicum* G1-33 showed good growth at pH 5.5–9.5 but minimal growth at pH 3.5 (Figure 1b). *E. acetylicum* G1-33 had good digestive enzyme activity at different pH levels, with that at pH 3.5–5.5 being the best (Figure S2). Growth results at different salinities showed that *E. acetylicum* G1-33 could grow at salinities ranging from 0 to 50 ppt. However, at different salinities, amylase activity was reduced, whereas lipase and protease activities increased (Figure 1c and Figure S2). The bile salt tolerance assay showed that *E. acetylicum* G1-33 could grow at a bile salt concentration of 0–0.3%, but its growth performance deteriorated with an increase in bile salt concentration (Figure 1d). The detection of digestive enzyme activity showed that *E. acetylicum* G1-33 exhibited amylase and lipase activities at bile salt concentrations of 0–3%, whereas protease activity was the highest at a bile salt concentration of 3% (Figure S2).

### 3.2. Growth Performance

The effects of *E. acetylicum* G1-33 supplementation on the growth performance of hybrid grouper are shown in Table 1. Compared with those of group C, the FW, WG, WGR, SGR, and VI of groups L and M increased, with a significant increase observed in group M (*p* < 0.05). However, the CF was the highest in group C and was significantly higher than that in groups L and H, with no significant difference from group M. Notably, the growth performance of group H was the worst, and growth was inhibited compared with that in group C. In addition, *E. acetylicum* G1-33 supplementation increased the SR of the hybrid grouper, most notably in group M; however, this increase was not significant compared with that in group C (*p* > 0.05).

**Figure 2:**
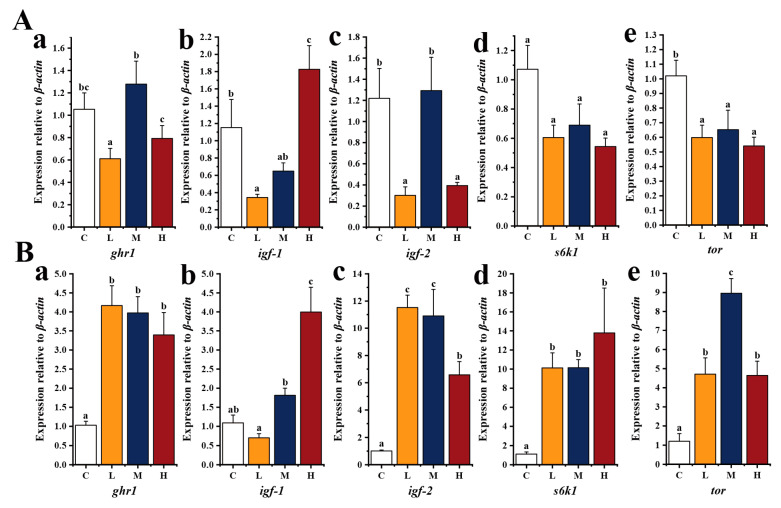
Relative expression of growth-related genes in liver (**A**) and muscle (**B**) of hybrid grouper (*E. fuscoguttatus* ♀ × *E. lanceolatus* ♂). C: Control, diet without *E. acetylicum* G1-33. L, M, H: diets supplemented with *E. acetylicum* G1-33 at levels of 10^6^, 10^8^, and 10^10^ CFU/g, respectively. *ghr1*: growth hormone receptor 1, *igf-1*: insulin-like growth factor-1, *igf-2*: insulin-like growth factor-2, *s6k1*: ribosomal protein S6 kinase 1, *tor*: target of rapamycin. Each value represents mean ± SD (n = 9). Bars with different superscripts significantly differ (*p* < 0.05, by one-way ANOVA).

To elucidate the potential mechanism by which *E. acetylicum* G1-33 improves the growth of hybrid grouper, the expression levels of growth-related genes in the liver and muscle were examined (Figure 2). The results showed that compared with those in group C, the expression levels of *ghr-1* and *igf-2* in the liver of hybrid grouper in group M were improved, while the expression levels of *igf-1* in group H were significantly increased (*p* < 0.05). Expression levels of *s6k1* and *tor* were lower in the *E. acetylicum* G1-33 supplementation groups than in group C. In the *E. acetylicum* G1-33 supplementation groups, the growth-related genes exhibited the highest expression in group M. In addition, compared with those in group C, *E. acetylicum* G1-33 supplementation significantly increased the expression levels of *ghr1*, *igf-2*, *s6k1*, and *tor* in fish muscle (*p* < 0.05). In the *E. acetylicum* G1-33 supplementation group, the expression of *ghr1* and *igf-2* was highest in group L, though no significant difference was observed between groups L and M (*p* > 0.05). The expression of *igf-1* and *s6k1* in group H was the highest, but only *ifg-1* expression was significantly higher than that in groups L and M (*p* < 0.05). In group M, *tor* expression was the highest, significantly higher than that in group L and group M (*p* < 0.05). 

### 3.3. Expression of Immune-Related Genes after the Feeding Program

**Figure 3:**
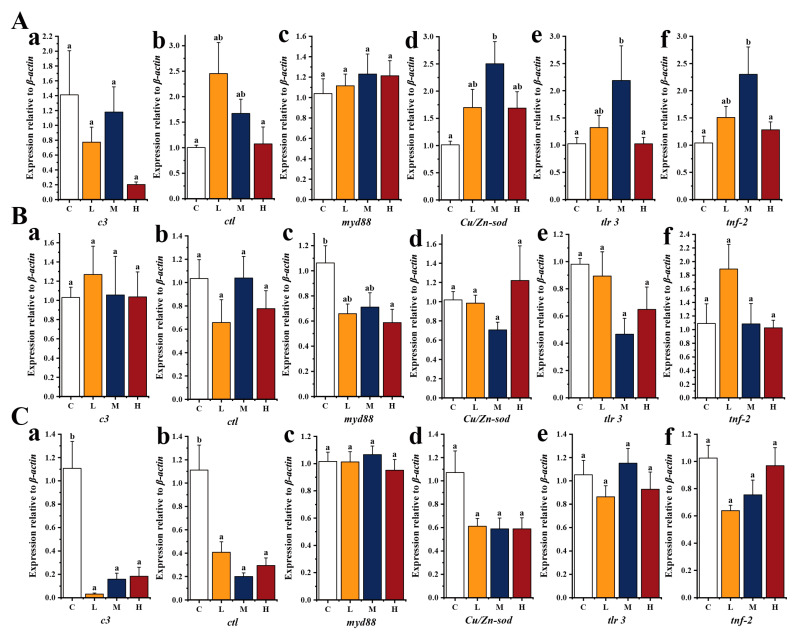
Relative expression levels of immune-related genes in head kidney (**A**), liver (**B**), and spleen (**C**) of hybrid grouper (*E. fuscoguttatus ♀* × *E. lanceolatus ♂*). C: control, diet without *E. acetylicum* G1-33. L, M, H: diets supplemented with *E. acetylicum* G1-33 at levels of 10^6^, 10^8^, and 10^10^ CFU/g, respectively. *c3*: complement C3, *ctl*: cytotoxic T lymphocytes, *myd88*: myeloid differentiation primary response gene 88, *Cu/Zn-sod*: Cu/Zn-superoxide dismutase, *tlr 3*: toll-like receptor 3, *tnf-2*: tumor necrosis factor-2. Each value represents mean ± SD (n = 9). Bars with different superscripts significantly differ (*p* < 0.05, by one-way ANOVA).

To investigate the effect of *E. acetylicum* G1-33 supplementation on the immunity of hybrid grouper, we examined the expression levels of immune-related genes in the head kidney, liver, and spleen (Figure 3). In the head kidney, the expression of *c3* in the *E. acetylicum* G1-33 supplementation groups was lower than that in group C, and the expression of other immune-related genes was higher than that in the group C. In the *E. acetylicum* G1-33 supplementation groups, the expression levels of *c3*, *myd88*, *Cu/Zn-sod*, *tlr 3*, and *tnf-2* were the highest in the M group, but only *tlr3* and *tnf-2* had significant differences (*p* < 0.05). The expression of *ctl* was highest in group L, and with increasing doses of *E. acetylicum* G1-33, *ctl* expression decreased gradually. In the liver, *c3* and *tnf-2* expression was the highest in group L. The expression levels of *ctl* and *Cu/Zn-sod* were the highest in groups M and H, respectively, but were not significantly different from those in group C (*p* > 0.05). The expression of *myd88* and *tlr 3* was the highest in group C, but no significant differences were observed between groups C, L, and M (*p* > 0.05). In the spleen, compared with that in group C, feeding *E. acetylicum* G1-33 reduced the expression of *c3*, *ctl*, *Cu/Zn-sod*, and *tnf-2*. Although the expression of *myd88* and *tlr 3* was the highest in group M, there was no significant difference compared with that in group C (*p* > 0.05). 

### 3.4. Biochemical Analysis

**Figure 4:**
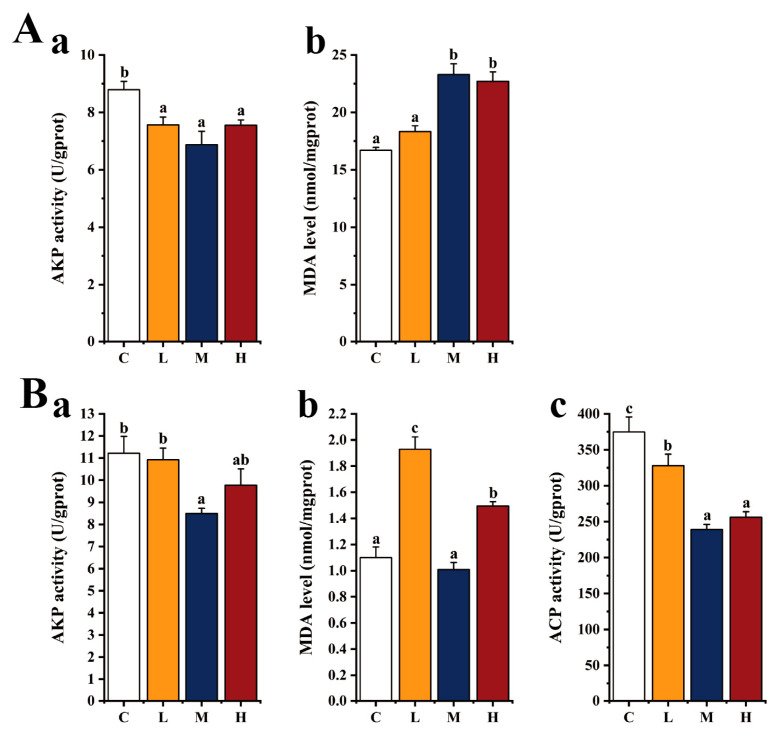
Immune-related enzyme activities in serum (**A**) and liver (**B**) of hybrid grouper (*E. fuscoguttatus* ♀ × *E. lanceolatus* ♂). C: control, diet without *E. acetylicum* G1-33. L, M, H: diets supplemented with *E. acetylicum* G1-33 at levels of 10^6^, 10^8^, and 10^10^ CFU/g, respectively. (**a**): AKP: alkaline phosphatase, (**b**): MDA: malondialdehyde, (**c**): ACP: acid phosphatase. Each value represents mean ± SD (n = 9). Bars with different superscripts significantly differ (*p* < 0.05, by one-way ANOVA).

Figure 4 shows the biochemical results of the liver and serum analyses after feeding *E. acetylicum* G1-33 to hybrid grouper. In the serum and liver, AKP activity was lower in the *E. acetylicum* G1-33 supplementation groups than in group C, though only significantly so in the liver (*p* < 0.05). With regards to the liver, ACP activity in groups M and H was significantly lower than that in group C (*p* < 0.05). Notably, the serum MDA content in the *E. acetylicum* G1-33 supplementation groups increased with increasing doses of *E. acetylicum* G1-33, being significantly higher in groups M and H than in group C (*p* < 0.05). The MDA content in the liver in group L was significantly higher than that of other groups (*p* < 0.05). And the MDA content in group M was lower than that in group C.

### 3.5. Histology

**Figure 5:**
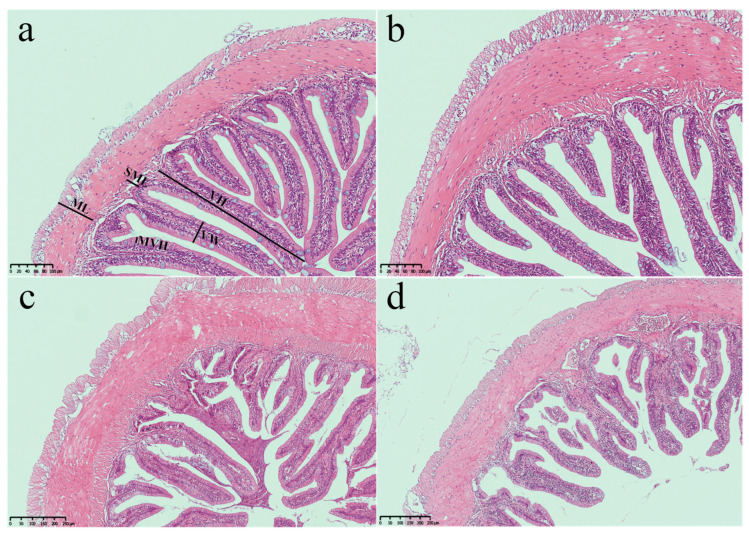
Intestinal tissue sections (100× magnification) of hybrid grouper (*E. fuscoguttatus* ♀ × *E. lanceolatus* ♂). Control (**a**), diet without probiotic. L (**b**), M (**c**), H (**d**): diets supplemented with probiotic at levels of 10^6^, 10^8^, and 10^10^ CFU/g, respectively. VW: villus width; VH: villus height; MVH: microvillus height; SML: submucosa layer thickness; ML: muscle layer thickness.

**Figure 6:**
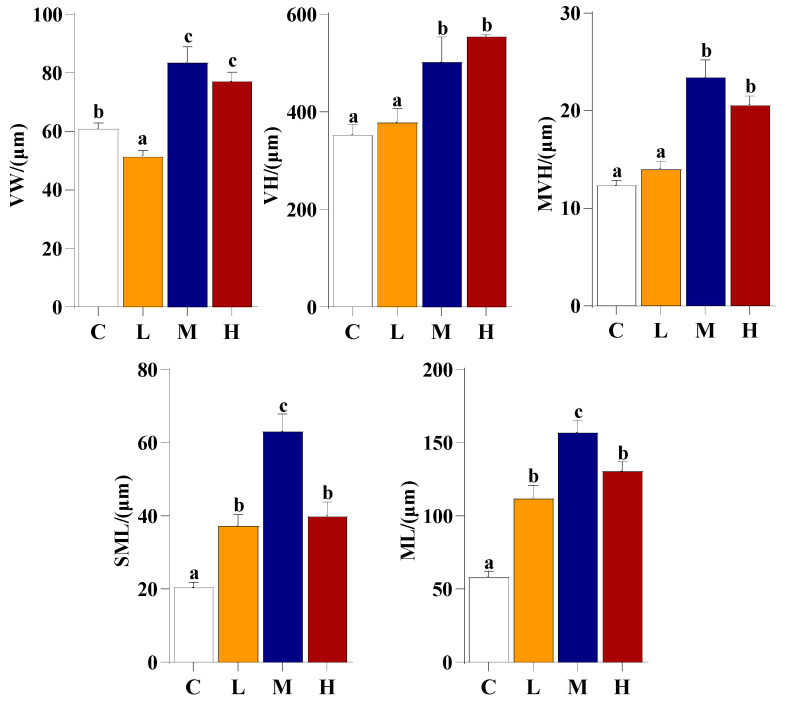
Morphological parameters of intestines of hybrid grouper (*E. fuscoguttatus* ♀ × *E. lanceolatus* ♂) after feeding *E. acetylicum* G1-33 for 60 days. Data are presented as mean ± SD (n = 9). Data with different letters are significantly different (*p* < 0.05, by one-way ANOVA). VW: villus width; VH: villus height; MVH: microvillus height; SML: submucosa layer thickness; ML: muscle layer thickness. C: control, diet without *E. acetylicum* G1-33. L, M, H: diets supplemented with *E. acetylicum* G1-33 at levels of 10^6^, 10^8^, and 10^10^ CFU/g, respectively.

The effect of administering *E. acetylicum* G1-33 on intestinal health was assessed by measuring intestinal morphology (Figure 5). The results showed that the villus height, microvillus height, submucosal layer thickness, and muscle layer thickness of the *E. acetylicum* G1-33 supplementation groups were higher than those of group C, with that of group M being the highest (*p* < 0.05). The villus width of group L was the lowest and was significantly lower than that of group C, whereas the villus widths of groups M and H were significantly higher than that of group C (*p* < 0.05, Figure 6). 

### 3.6. Hybrid Group SR after V. harveyi Challenge

**Figure 7:**
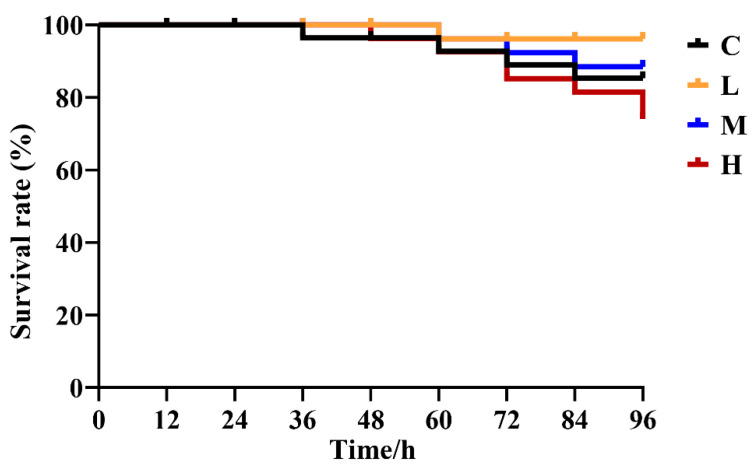
Survival rate of hybrid grouper (*E. fuscoguttatus* ♀ × *E. lanceolatus* ♂) after challenge with *V. harveyi*. C: control, diet without *E. acetylicum* G1-33. L, M, H: diets supplemented with *E. acetylicum* G1-33 at levels of 10^6^, 10^8^, and 10^10^ CFU/g, respectively.

After challenge with *V. harveyi*, death occurred in group C at 36 h and in the *E. acetylicum* G1-33 supplementation groups at 48 h (Figure 7). Compared with that in group C, a reduced mortality rate was observed in groups L and M after infection with *V. harveyi*, whereas reduced resistance was observed in group H. Of the three supplementation groups, group L exhibited the lowest mortality rate and the highest protective effect against *V. harveyi*.

### 3.7. Expression of Immune-Related Genes after V. harveyi Challenge

**Figure 8:**
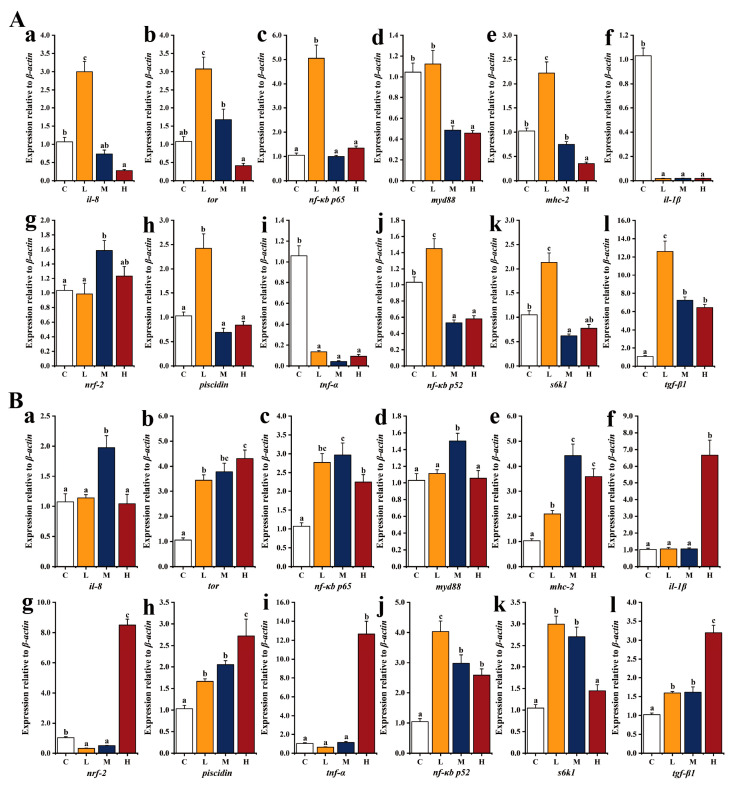
Relative expression levels of immune-related genes in head kidney of hybrid grouper (*E. fuscoguttatus* ♀ × *E. lanceolatus* ♂) after *V. harveyi* challenge for 48 h (**A**) and 96 h (**B**). C: control, diet without *E. acetylicum* G1-33. L, M, H: diets supplemented with *E. acetylicum* G1-33 at levels of 10^6^, 10^8^, and 10^10^ CFU/g, respectively. *il-8*: interleukin-8; *tor*: target of rapamycin; *nf-κb p65*: nuclear factor kappa B p65; *myd88*: myeloid differentiation primary response gene 88; *mhc-2*: major histocompatibility complex-2; *il-1β*: interleukin-1beta; *nrf-2*: nuclear factor erythoid 2-related factor 2; *tnf-α*: tumor necrosis factor alpha; *nf-κb p52*: nuclear factor kappa B p52; *s6k1*: S6 kinase 1; *tgf-β1*: transforming growth factor beta 1. Each value represents mean ± SD (n = 9). Bars with different superscripts significantly differ (*p* < 0.05, by one-way ANOVA).

**Figure 9:**
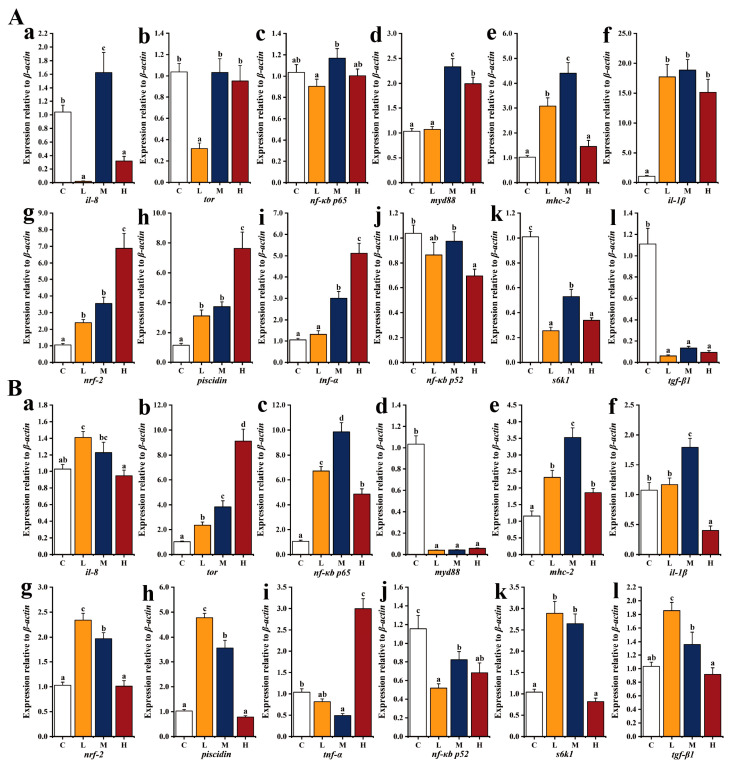
Relative expression levels of immune-related genes in liver of hybrid grouper (*E. fuscoguttatus* ♀ × *E. lanceolatus* ♂) after *V. harveyi* challenge for 48 h (**A**) and 96 h (**B**). C: control, diet without *E. acetylicum* G1-33. L, M, H: diets supplemented with *E. acetylicum* G1-33 at levels of 10^6^, 10^8^, and 10^10^ CFU/g, respectively. *il-8*: interleukin-8; *tor*: target of rapamycin; *nf-κb p65*: nuclear factor kappa B p65; *myd88*: myeloid differentiation primary response gene 88; *mhc-2*: major histocompatibility complex-2; *il-1β*: interleukin-1beta; *nrf-2*: nuclear factor erythoid 2-related factor 2; *tnf-α*: tumor necrosis factor alpha; *nf-κb p52*: nuclear factor kappa B p52; *s6k1*: S6 kinase 1; *tgf-β1*: transforming growth factor beta 1. Each value represents mean ± SD (n = 9). Bars with different superscripts significantly differ (*p* < 0.05, by one-way ANOVA).

To elucidate the resistance mechanism of an *E. acetylicum* G1-33-supplemented diet following *V. harveyi* challenge, the expression of genes related to immune performance in the head kidney and liver was examined (Figure 8 and Figure 9). After 48 h of challenge, the expression of genes, excluding that of *il-1β*, *nrf-2*, and *tnf-α*, in group L was significantly higher than that in group C in the head kidney (*p* < 0.05). The expression of most of the detected genes in groups M and H was lower than that in group C, with only *nrf-2* and *tgf*-*β1* being higher than those in the C group (Figure 8A). At 96 h, excluding that of *nrf-2* and *tnf-α*, the expression of genes in the *E. acetylicum* G1-33 supplementation groups was higher than that in group C: *il-8*, *nf-κb p65*, *myd88*, and *mhc-2* had the highest expression in group M; *tor*, *il-1β*, *nrf-2*, *piscidin*, *tnf-α*, and *tgf-β1* had the highest expression in group H; and *nf-κb p52* and *s6k1* had the highest expression in group L, with values significantly higher than those in group C (*p* < 0.05, Figure 8B).

In contrast to the gene expression patterns in head and kidney, 48 h following challenge, most of the detected genes in the liver had the highest expression in group M, with the exception of *nrf-2*, *piscidin*, and *tnf-α*, which were the highest in group H, and the difference was significant (*p* < 0.05, Figure 9A). At 96 h, compared with that in group C, the expression of most detected genes was higher in groups L and M, and the expression of *tor*, *nf-κb p65*, *mhc-2*, and *tnf-α* was higher in group H (Figure 9B).

## 4. Discussion

The occurrence of infectious diseases has emerged as a primary impediment to the further development of mariculture during its intensive expansion. The use of antibiotics and other chemicals has become a cost-effective solution to this problem. Lulijwa et al. [27] reported that 67 antibiotics were used in the top 15 aquaculture-producing countries between 2008 and 2018, with the majority of fishers using antibiotics for disease prevention and treatment without prescription, toxicology, or environmental impact assessment. It is worth noting that the excessive and inappropriate use of antibiotics can result in adverse effects on fish, the environment, and human health [28]. Because antibiotics used in fish farming are not fully digested and absorbed, they are released into coastal waters through urine and feces and can be persistent [29]. Li et al. [30] reported that in China, due to the extensive use of antibiotics in aquaculture, 33 antibiotics with different concentrations were distributed in China’s coastal waters. As such, the use of probiotics to regulate water microorganisms, enhance the immunity of farmed fish, and improve aquaculture yields has become one of the most widely accepted strategies for control and prevention in mariculture. In mariculture, probiotics are predominantly administered via immersion and oral routes, and the effectiveness of oral administration has been confirmed in species such as *E. coioides* and *Pagrus major* [6,31]. Probiotics that work orally must resist transport in the gastrointestinal tract of fish, necessitating survival under low-pH and bile conditions [32]. In this study, *E. acetylicum* G1-33 grew at pH values ranging from 5.5 to 9.5 and bile salt concentrations ranging from 0 to 0.3%, indicating its tolerance. Although its growth was limited at pH 3.5 and a bile salt concentration of 3%, when taken orally, probiotics are coated with feed, potentially enhancing their tolerance to low-pH and high-bile-salt conditions. Additionally, a suitable temperature and salinity are crucial for the successful application of *E. acetylicum* G1-33 in hybrid grouper cultures [33]. As a tropical marine fish, the grouper grows within specific temperature and salinity ranges; studies have shown that the average feeding temperature of grouper is 26.32 ± 0.62 °C, and it can grow and survive in a salinity range of 10–30 ppt, aligning with the adapted growth conditions of *E. acetylicum* G1-33 [34]. In addition, *E. acetylicum* G1-33 demonstrates strong extracellular enzymatic activity, indicating that it could make better use of nutrients to promote host growth. Studies have shown that enzymes produced by gut bacteria can improve digestion and absorption processes by facilitating the breakdown of food, which, in turn, has a positive impact on the host [35]. In general, our findings indicate that *E. acetylicum* G1-33 has high application potential in the grouper culture industry.

Growth improvement is an important index for evaluating the effects of probiotics. The results of this study showed that supplementation with *E. acetylicum* G1-33 at 10^6^ and 10^8^ CFU/g can improve the growth of hybrid grouper, with 10^8^ CFU/g showing the most significant effect. Notably, we observed that supplementation with *E. acetylicum* G1-33 at 10^10^ CFU/g significantly inhibited the growth of the hybrid grouper. Shen et al. [36] found that feeding *L. vannamei* with *Bacillus subtilis* at concentrations of 1 × 10^4^ and 5 × 10^4^ CFU/g could improve the growth of this shrimp, but growth was not promoted at a dose of 1 × 10^5^ CFU/g. Similar results have been reported in *Poecilia sphenops* and *Xiphophorus helleri*; at 5 × 10^5^–5 × 10^7^ CFU/g, the growth promotion effect of *B. subtilis* enhances as its concentration increases, until the concentration reaches 5 × 10^8^ CFU/g, at which point growth is inhibited [37]. The reduced growth caused by a higher proportion of probiotics may be due to an imbalance in the gastrointestinal microbial community and indigestion caused by microbial invasion. Therefore, choosing an appropriate dose of probiotics to avoid excessive or low doses is essential, as making an improper choice could result in financial losses.

Regulating the growth of fish is a complex process. The growth hormone (GH)–IGF axis is the primary mechanism controlling growth and tissue proliferation in vertebrates, including fish [38]. In fish, GHR1 and GHR2, which are important GH-binding receptors, are expressed in the liver and muscle and can mediate the action of GH. The activation of these receptors can lead to the activation of IGF signal transduction and regulate cell proliferation and morphological parameters in fish [39]. Furthermore, IGF-1 can regulate TOR transcription and expression. The TOR signaling pathway is involved in protein metabolism and growth and regulates protein synthesis through S6K1 and eukaryotic translation initiation factor 4E-binding protein (4E-BP) [40]. The TOR signaling pathway and GH–IGF axis often collaborate to regulate fish growth [41]. Jose et al. [42] found that administering a combination of the probiotics *Paenibacillus polymyxa* HGA4C and *Bacillus licheniformis* HGA8B could upregulate the expression of *ghr1*, *ghr2*, *ifg1*, and *ifg2* in the liver and muscle of *O. niloticus* and improve its growth rate. Heshmatfar et al. [43] confirmed that the supplementation of *Pediococcus acidilactici* could significantly enhance the growth performance of *Cyprinus carpio*, which was correlated with a substantial upregulation in *igf-1* expression. The level of *igf-1* expression in the supplementation group was approximately five times higher compared to that in the control group. Li et al. [44] reported that a *Clostridium butyricum* diet could activate the expression of genes related to the TOR signaling pathway in *L. vannamei*, resulting in an increase in the final weight and specific growth rate, as well as a significant decrease in the feed conversion ratio, of shrimp. Moreover, Liang et al. [45] and Irm et al. [46] found that TOR signaling pathway activation and downstream regulators S6K1 and 4E-BP were closely related to growth in *Megalobrama amblycephala* and *Acanthoparus schlegelii*. These findings align with those of the current study, which revealed that a diet containing *E. acetylicum* G1-33 could modulate the expression of growth-related genes in the muscle and liver of hybrid grouper, thereby regulating their growth. Notably, gene expression levels in the liver were lower than those in the muscle, which may be caused by the negative regulation of genes, necessitating further investigations to elucidate the underlying mechanisms.

Previous studies have highlighted the ability of probiotics to produce enzymes capable of effectively metabolizing various lipids, proteins, and carbohydrates, thereby improving feed utilization and fish growth [47]. In the present study, *E. acetylicum* G1-33 secreted a diverse range of digestive enzymes under different conditions, enhancing the decomposition, digestion, and absorption of nutrients in the gut of hybrid grouper, consequently promoting growth [48]. Additionally, the gut is the primary site of nutrient absorption, and intestinal morphology is closely related to fish growth [49]. This study found that administering *E. acetylicum* G1-33 did not adversely affect the important internal organs of the hybrid group. At 10^6^ and 10^8^ CFU/g, *E. acetylicum* G1-33 improved intestinal villus width, villus height, and microvillus height. This optimization of intestinal morphology can increase the intestinal absorption area and promote the effective absorption of nutrients. Since the proportion of villi is related to the ability to absorb nutrients through the available surface area, the surface increase could potentially improve nutrient utilization and storage, thus promoting growth [50]. In *O. niloticus*, a *Lactobacillus rhamnosus* diet was confirmed to be able to cause a significant increase in villus height, which implies an increase in surface area for better absorption of available nutrients [51]. Merrifield et al. [52] demonstrated in vivo that *P. acidilactici* was able to enhance the enterocyte microvilli of *O. mykiss*, which would directly increase the absorption surface area of the gut. However, supplementation with 10^10^ CFU/g *E. acetylicum* G1-33 inhibited the development of intestinal microvilli, which may explain why the growth of the hybrid grouper was inhibited. In general, the addition of 10^8^ CFU/g *E. acetylicum* G1-33 can improve the intestinal morphology and absorption capacity of fish.

The immunomodulatory effect is a well-documented benefit of probiotics in aquaculture [53]. Within fish, the innate immune system serves as the primary defense mechanism against pathogen invasion, functioning mainly via the pattern recognition receptors (PRRs). Among these, the toll-like receptor (TLR) family plays a crucial role by identifying pathogen-associated molecular patterns, thereby initiating downstream signaling pathways involving NF-κB, interferon modulators, and other regulatory molecules via MyD88-dependent or -independent immune signaling pathways [8,54,55]. Studies have shown that TLR3 can induce cytokine release through a MyD88-independent pathway, whereas MyD88-dependent pathway activation by other TLRs leads to the recruitment of TNF receptor-associated factor 6 (TRAF6) and the subsequent promotion of proinflammatory cytokine expression [56]. Dang et al. [8] found that probiotics upregulate the expression of *tlrs* (1, 2, 3, 4, 5, 7, 9, and 22) and signaling cascade genes of the TLR pathway (i.e., *myd88* and *tnf-α*) in *O. mykiss*, resulting in reduced mortality during *Aeromonas salmonicida* infection. Furthermore, studies on *Channa argus*, common carp, and *Pelteobagrus fulvidraco* have shown that superoxide dismutase (SOD), C3, and CTL play important roles in protecting fish health and can improve resistance to *A. hydrophila*, *A. veronii*, and *Edwardsiella ictalurid* [57,58,59]. The addition of 10^8^ CFU/g *E. acetylicum* G1-33 in the current study improved the expression of *tlr3*, *myd88*, *ctl*, and other genes in the head kidney and liver of hybrid grouper, consistent with the observations of aforementioned studies. These findings indicate that supplementation with *E. acetylicum* G1-33 improves the innate immunity of hybrid grouper, particularly of the liver and head kidney, though weakened immunity of the spleen was noted, and enhances their resistance to pathogens. Notably, an *E. acetylicum* G1-33-supplemented diet led to an increase in MDA content and a decrease in AKP and ACP activities in the liver and serum of the hybrid grouper. MDA content reflects peroxidative tissue damage and is an important marker of oxidative stress, with an elevated MDA content indicating high oxidative stress, which is unfavorable for fish health. AKP and ACP are lysosomal enzymes that play important roles in resisting pathogen invasion [60]. Studies have shown that the MDA content in the gut of Pengze crucian carp decreases and the activities of AKP and ACP increase after feeding these fish *B. cereus*, indicating a positive effect on intestinal immunity [61,62]. 

To further assess the effect of an *E. acetylicum* G1-33-supplemented diet on disease resistance in hybrid grouper, a pathogen challenge test with *V. harveyi* was performed following the feeding experiment. Pathogen challenge tests are commonly used to assess fish health following dietary management [63]. *V. harveyi* is a gram-negative bacterium capable of causing severe diseases in a variety of mariculture fish, including grouper [14]. Grouper infected with *Vibrio* spp. show symptoms such as a loss of appetite, gill and body surface damage, abnormal swimming posture, and weakened exercise ability and are usually co-infected with other bacteria and viruses, which can bring huge economic losses to grouper aquaculture [64]. Several studies have demonstrated that a probiotic diet can enhance the resistance of grouper to *V. harveyi* [19,65], which is consistent with the findings of the present study. In the present study, we supplemented the diet of hybrid grouper with 10^6^ and 10^8^ CFU/g of *E. acetylicum* G1-33, which resulted in reduced *V. harveyi*-induced mortality. However, the resistance of the hybrid grouper to *V. harveyi* notably decreased and mortality increased when *E. acetylicum* G1-33 was administered at a concentration of 10^10^ CFU/g. The reason for these results may be that activation of the host immune system is related to the dose and type of probiotics. Amoah et al. [65] found that the resistance of grouper to *V. harveyi* was significantly influenced by probiotic species when different probiotics were fed at the same dose. Similarly, Tan et al. [66] observed that probiotic supplementation affected the resistance of *O. niloticus* to various pathogens; however, higher doses of probiotics did not necessarily increase this resistance. These results were confirmed by measuring the expression of immune-related genes. In the present study, immune-related genes (*il-8*, *tor*, *nf-κb p65*, *myd88*, *mhc-2*, *piscidin*, etc.) were extensively upregulated in the head kidney of group L 48 h after *V. Harvey* infection, whereas the upregulation of immune-related genes was not detected until 96 h after infection in groups M and H. MyD88 is an important transduction protein that regulates adaptive immunity-related pathways, mediates the activation of most TLRs, and induces the activation of interferon regulatory factors [67]. The NF-κB p65 signaling pathway is closely related to the level of inflammation, and p65 plays an important role in regulating the inflammatory response [68]. IL-8 is a key immune factor in inducing cytokines and defending against pathogen infection, plays a key role in inflammation regulation, and is one of the important parameters in evaluating the innate immune system [69]. TOR is not only involved in the regulation of protein synthesis but also plays an important role in the control of inflammatory responses [70]. MCH-2 plays an important role in antigen presentation and pathogen recognition and is able to activate innate immunity [71]. Piscidin family proteins are an important part of the innate immune system of lower vertebrates, showing strong broad-spectrum activity against bacteria, fungi, parasites, and even viruses [72]. In conclusion, supplementing the diet of hybrid grouper with *E. acetylicum* G1-33 at 10^6^ or 10^8^ CFU/g enhances disease resistance; however, the regulatory mechanism between probiotic dose and host disease resistance needs to be further elucidated.

## 5. Conclusions

In vitro evaluation confirmed that *E. acetylicum* G1-33 has multiple probiotic properties and is a potential probiotic strain. The subsequent hybrid grouper feeding study showed that diets containing 10^6^ and 10^8^ CFU/g of *E. acetylicum* G1-33 significantly improved the growth performance of hybrid grouper, which may be related to the upregulation of growth-related genes in muscle and the positive impact of this probiotic on intestinal morphology. The diet supplemented with *E. acetylicum* G1-33 also modulated the expression of multiple immune-related genes, which may improve the immunity of hybrid grouper. In addition, this study showed that diets supplemented with 10^6^ and 10^8^ CFU/g of *E. acetylicum* G1-33 reduced the mortality of hybrid grouper after *V. harveyi* infection, which may be related to the rapid activation of the immune system; however, a diet supplemented with 10^10^ CFU/g of *E. acetylicum* G1-33 reduced disease resistance in hybrid grouper. Therefore, further studies are needed to elucidate the regulatory mechanism between probiotic dose and host disease resistance. In conclusion, this study demonstrates the potential of *E. acetylicum* G1-33 as a probiotic in mariculture, offering multifaceted benefits including enhanced growth, improved immunity, disease mitigation, and organ safety. The best results can be obtained when the G1-33 dosage is 10^8^ CFU/g.

## Figures and Tables

**Table 1:** Growth performance and survival rate of hybrid grouper (*E. fuscoguttatus* ♀ × *E. lanceolatus* ♂) after feeding *E. acetylicum* G1-33 for 60 days.

	FW (g)	WG (g)	WGR (%)	SGR (%)	CF (%)	VI (%)	SR (%)
C	65.17 ± 1.51 ^b^	34.97 ± 1.52 ^b^	52.10 ± 1.15 ^b^	1.25 ± 0.04 ^b^	2.97 ± 0.06 ^a^	6.35 ± 0.13 ^ab^	94.44 ± 1.11 ^a^
L	67.02 ± 1.72 ^b^	36.88 ± 1.74 ^b^	53.08 ± 1.32 ^b^	1.30 ± 0.05 ^b^	2.76 ± 0.05 ^b^	6.43 ± 0.12 ^a^	95.56 ± 1.11 ^a^
M	71.16 ± 1.57 ^a^	41.16 ± 1.57 ^a^	56.57 ± 0.99 ^a^	1.41 ± 0.04 ^a^	2.87 ± 0.05 ^ab^	6.51 ± 0.17 ^a^	98.89 ± 1.11 ^a^
H	57.36 ± 1.59 ^c^	28.70 ± 1.52 ^c^	48.03 ± 1.35 ^c^	1.12 ± 0.04 ^c^	2.75 ± 0.06 ^b^	6.11 ± 0.18 ^b^	94.44 ± 1.11 ^a^

Data are presented as mean ± SD (n = 3). Data with different letters are significantly different (*p* < 0.05, by one-way ANOVA). FW: final weight; WG: weight gain; WGR: weight gain rate; SGR: specific growth rate; CF: condition factor; VI: visceral index; SR: survival rate. C: Control, diet without *E. acetylicum* G1-33. L, M, H: diets supplemented with *E. acetylicum* G1-33 at the levels 10^6^, 10^8^, and 10^10^ CFU/g, respectively.

## Data Availability

The original contributions presented in the study are included in the article/Supplementary Material, further inquiries can be directed to the corresponding author.

## Supplementary Materials

The following supporting information can be downloaded at: https://www.mdpi.com/article/10.3390/microorganisms12081688/s1, Figure S1: Colonies, Gram staining, and SEM photograph of *Exiguobacterium acetylicum* G1-33; Figure S2: Amylase (a), lipase (b), and protease (c) activities of *Exiguobacterium acetylicum* G1-33 under different temperature, pH, salinity, and bile salt conditions. Each value represents mean ± SD (n = 3). Bars with different superscripts significantly differ (*p* < 0.05, by one-way ANOVA); Table S1: Proximate composition of the commercial feed; Table S2: Primers and amplification conditions used for quantitative RT-PCR (qPCR). References [71,73,74,75,76,77,78] are cited in the Supplementary Materials.
