# Supplementary material for: Host Gut-Derived Probiotic, Exiguobacterium acetylicum G1-33, Improves Growth, Immunity, and Resistance to Vibrio harveyi in Hybrid Grouper (Epinephelus fuscoguttatus ♀ × Epinephelus lanceolatus ♂)
Source: Microorganisms. 2024 Aug 16;12(8):1688. doi: 10.3390/microorganisms12081688 (PMC11357496; doi:10.3390/microorganisms12081688)
Supplement: Supplementary file 1 [file microorganisms-12-01688-s001.zip › Tables S1 and S2.pdf]

Table S1. Proximate composition of commercial feed.

**Nutrients**	**Percent (%)**
Crude fiber	≤5.0
Crude protein	≥49.0
Crude fat	≥8.0
Lysine	≥2.5
Total phosphorus	≥1.5
Crude ash	≤16.0
Moisture	≤12.0

Table S2. Primers and amplification conditions used for quantitative RT-PCR (qPCR).

Target Genes	Primers Sequences (5′-3′) for qPCR	Annealing Temperature (°C) for qPCR	Reference
β-actin	F: GACATCAAGGAGAAGCTGTG<br>R: TGCTGTTGTAGGTGGTCTCGT	60	[1]
tor	F: CCACTCTTTCTTTGCGGCTT<br>R: GGGTTCTCGTCCCTCACTTG	60	[2]
s6k1	F: TCCTTCTCCGTCTGTAAACGA<br>R: CATGAACACCTGCTTACCAT	60	[3]
ghr1	F: CACAGACTTCTATGCCCAGGT<br>R: GTGTAGCCGCTTCCTTCAG	60	[3]
igf-1	F: ACCCCTGTGGAATGGTTCAC<br>R: TCCCGATATGGAATGGTGCAG	60	[4]
igf-2	F: GTGATGCCCGCACTAAAACC<br>R: TCAGGGACATAGGGGTGAGG	60	[4]
Cu/Zn-sod	F: CCCCACAACAAGCAGC<br>R: GCAACATTATCGCCTCC	60	[5]
myd88	F: AGCTGGAGCAGACGGAGTG<br>R: GAGGCTGAGAGCAAACTTGGTC	60	[6]
ctl	F: GGCAAGATGGGAGCCTAAC<br>R: ATGCGACGAATGTCCTGAA	60	[5]
c3	F: CGGTTGGCAGAGGTAGTGGA<br>R: GGTGTTTTCGTGAAGTGAATGGT	60	[5]
tnf-2	F: ACACTCTGCCTCGCCTCTG<br>R: CCTGACTCCTCCATTCCACTG	60	[5]
tlr 3	F: CTGGCTTACTACAACCACCCC<br>R: CAAACTCCCTGCCCTCTTCA	60	[6]

il-1β	F: ACCCACGTCCACCTCCATGA<br>R: TTGCTTTCCCGGCTGCAGAA	58	
il-8	F: CATGATGGAAGCCATGAG<br>R: GTCAGCGTGTCAGGAAT	60	
nrf-2	F: GAAGGAGCGTCTGTTGAGTGA<br>R: GAAGATGCTGCCGTTAGTTGA	58	
nf-κb p65	F: TCCAGGCCTCCATCACTCTG<br>R: CATAGATGGGCTGCGACACC	60	
nf-κb p52	F: GGCAGCAAGAGAGCTGGAAG<br>R: GCAACACAGCGCGTTCATAC	60	
piscidin	F: GATCATCCTGCCAGCGTGAA<br>R: GTGCATCGCGCTCTTTCTTG	58	
tnf-α	F: GCCACAGGATCTGGCGCTACTC<br>R: CTTCCGTCGCTGTCCTCATGTG	58	
mhc-2	F: CCACCCGAACAAACAGACC<br>R: TGATGCCCCCTCCAACACT	58	[7]
tgf-β1	F: AACATCCCGCTACCTCGCTT<br>R: TCCGCTCATCCTCATTCCCT	58	[7]

tor: target of rapamycin; *s6k1*: ribosomal protein s6 kinase 1; *ghr1*: growth hormone receptor 1; *igf-1*: insulin-like growth factor-1; *igf-2*: insulin-like growth factor-2; *myd88*: myeloid differentiation primary response gene 88; *ctl*: cytotoxic T lymphocytes; *c3*: complement 3; *tnf-2*: tumor necrosis factor-2; *tlr 3*: toll-like receptor 3; *il-1β*: interleukin-1beta; *nrf-2*: nuclear factor erythoid 2-related factor 2; *nf-κb p52*: nuclear factor kappa B p52; *tgf-β1*: transforming growth factor beta 1; *mhc-2*: major histocompatibility complex-2; *tnf-α*: tumor necrosis factor alpha; *il-8*: interleukin-8; *nf-κb p65*: nuclear factor kappa B p65.

References

1. Yang, H.-L.; Sun, Y.-Z.; Hu, X.; Ye, J.; Lu, K.-L.; Hu, L.-H.; Zhang, J.-J. Bacillus Pumilus SE5 Originated PG and LTA Tuned the Intestinal TLRs/MyD88 Signaling and Microbiota in Grouper (Epinephelus Coioides). *Fish & Shellfish Immunology* **2019**, *88*, 266–271. https://doi.org/10.1016/j.fsi.2019.03.005
2. Tan, X.; Sun, Z.; Liu, Q.; Ye, H.; Zou, C.; Ye, C.; Wang, A.; Lin, H. Effects of Dietary Ginkgo Biloba Leaf Extract on Growth Performance, Plasma Biochemical Parameters, Fish Composition, Immune Responses, Liver Histology, and Immune and Apoptosis-Related

Genes Expression of Hybrid Grouper (*Epinephelus Lanceolatus*♂ × *Epinephelus Fuscoguttatus*♀) Fed High Lipid Diets. *Fish & Shellfish Immunology* **2018**, *72*, 399–409. https://doi.org/10.1016/j.fsi.2017.10.022
3. Zhou, Z.; Wang, X.; Wu, X.; Gao, Y.; Li, X.; Dong, Y.; Yao, W. Effects of Dietary Leucine Levels on Growth, Feed Utilization, Neuro-Endocrine Growth Axis and TOR-Related Signaling Genes Expression of Juvenile Hybrid Grouper (Epinephelus Fuscoguttatus ♀ × Epinephelus Lanceolatus ♂). *Aquaculture* **2019**, *504*, 172–181. https://doi.org/10.1016/j.aquaculture.2019.02.005
4. Xia, Y.-T.; Wu, Q.-Y.; Hok-Chi Cheng, E.; Ting-Xia Dong, T.; Qin, Q.-W.; Wang, W.-X.; Wah-Keung Tsim, K. The Inclusion of Extract from Aerial Part of *Scutellaria baicalensis* in Feeding of Pearl Gentian Grouper (*Epinephelus fuscoguttatus*♀ × *Epinephelus lanceo-latus*♂) Promotes Growth and Immunity. *Fish & Shellfish Immunology* **2022**, *127*, 521–529. https://doi.org/10.1016/j.fsi.2022.06.041
5. Luo, S.-W.; Cai, L.; Qi, Z.-H.; Wang, C.; Liu, Y.; Wang, W.-N. Effects of a Recombinant Complement Component C3b Functional Fragment α2MR (α2-macroglobulin receptor) Additive on the Immune Response of Juvenile Orange-Spotted Grouper (*Epinephelus coioides*) after the Exposure to Cold Shock Challenge. *Fish & Shellfish Immunology* **2015**, *45*, 346–356. https://doi.org/10.1016/j.fsi.2015.04.019
6. Lin, K.; Zhu, Z.; Ge, H.; Zheng, L.; Huang, Z.; Wu, S. Immunity to Nervous Necrosis Virus Infections of Orange-Spotted Grouper (*Epinephelus coioides*) by Vaccination with Virus-like Particles. *Fish & Shellfish Immunology* **2016**, *56*, 136–143. https://doi.org/10.1016/j.fsi.2016.06.056
7. Sun, Z.; Tan, X.; Ye, H.; Zou, C.; Ye, C.; Wang, A. Effects of Dietary *Panax notoginseng* Extract on Growth Performance, Fish Composition, Immune Responses, Intestinal Histology and Immune Related Genes Expression of Hybrid Grouper (*Epinephelus lanceolatus* ♂ × *Epinephelus fuscoguttatus* ♀) Fed High Lipid Diets. *Fish & Shellfish Immunology* **2018**, *73*, 234–244. https://doi.org/10.1016/j.fsi.2017.11.007
