# Supplementary figures and images for: Host Gut-Derived Probiotic, Exiguobacterium acetylicum G1-33, Improves Growth, Immunity, and Resistance to Vibrio harveyi in Hybrid Grouper (Epinephelus fuscoguttatus ♀ × Epinephelus lanceolatus ♂)
Source: Microorganisms. 2024 Aug 16;12(8):1688. doi: 10.3390/microorganisms12081688 (PMC11357496; doi:10.3390/microorganisms12081688)

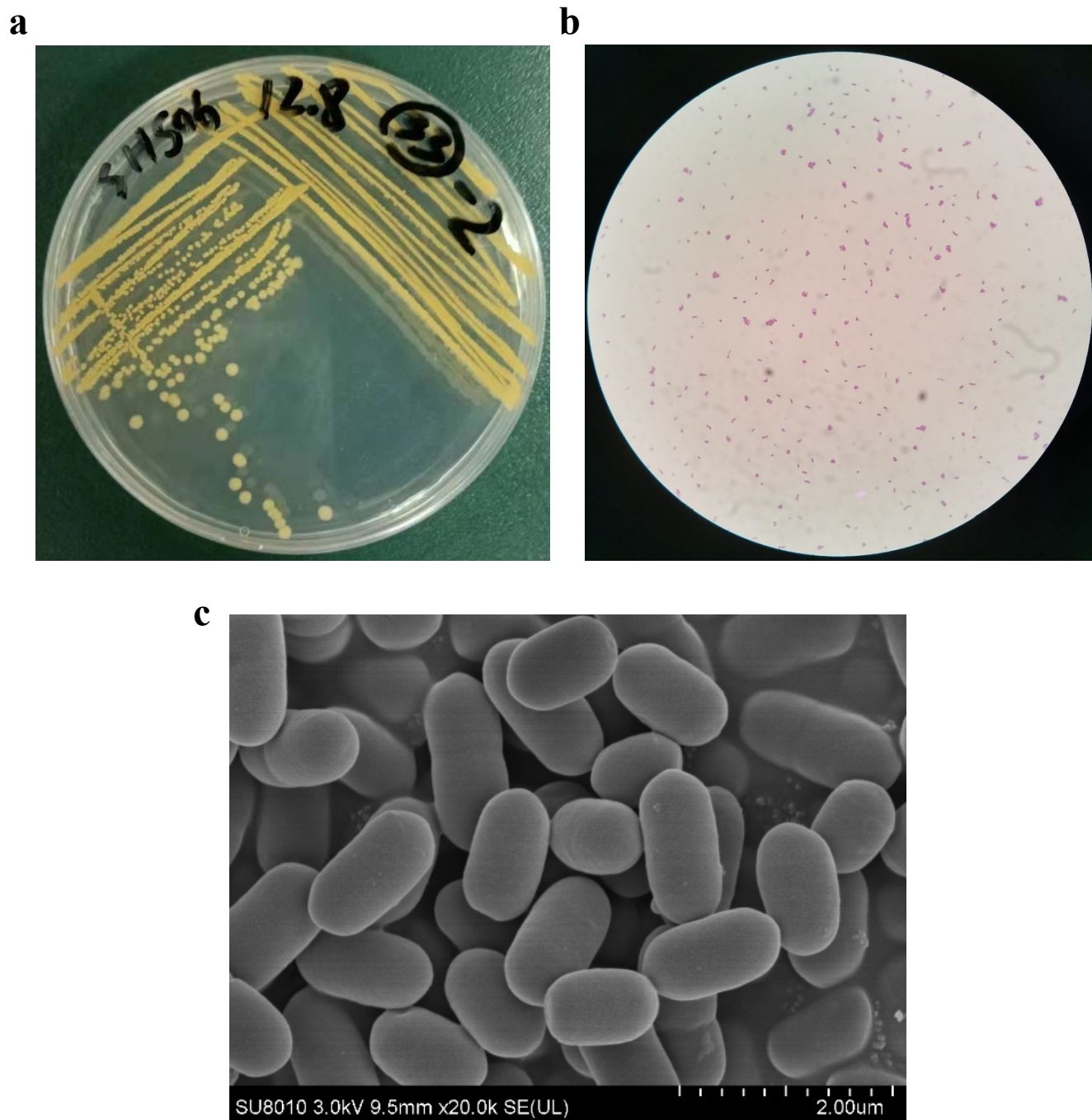

**Figure S1**

Supplement: Supplementary file 1 [file microorganisms-12-01688-s001.zip › Figure S1.pdf]

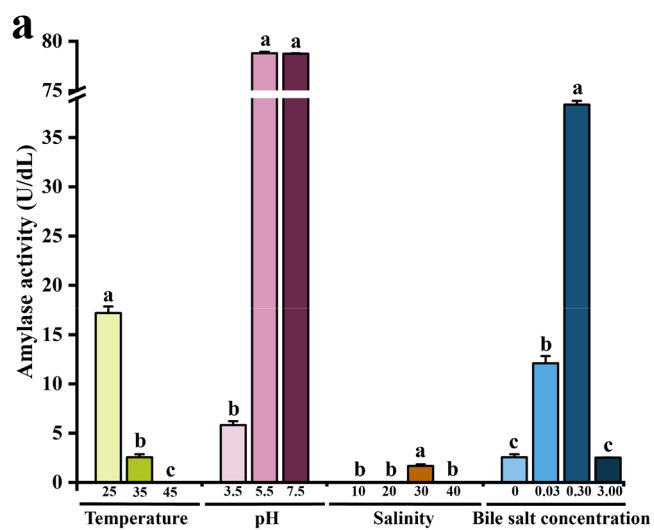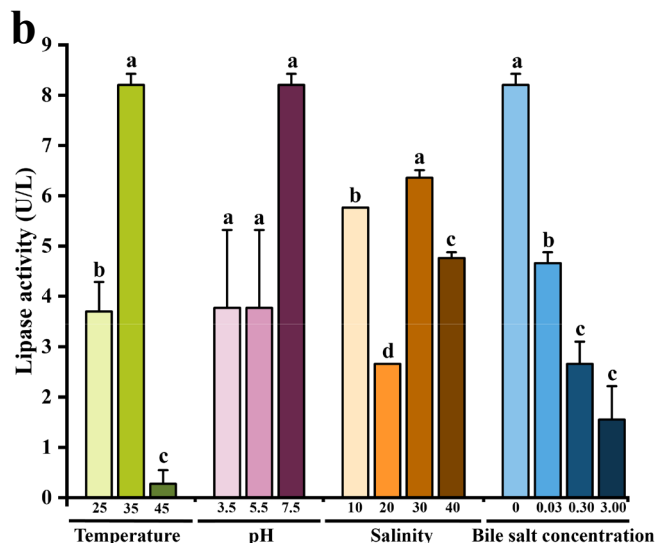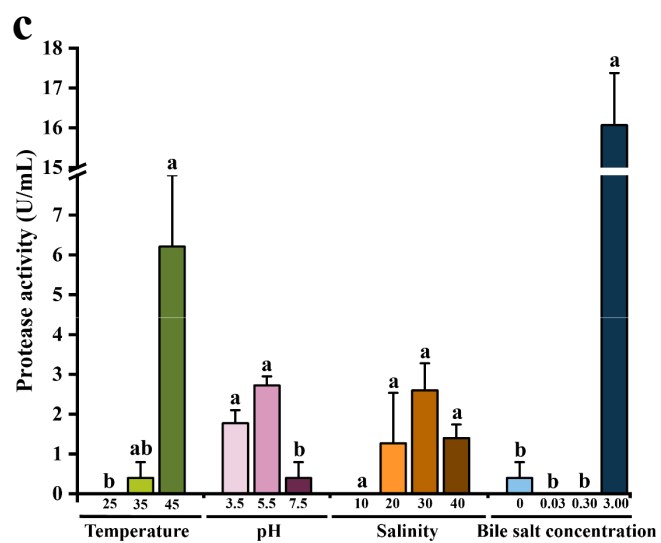

**Figure S2**

Supplement: Supplementary file 1 [file microorganisms-12-01688-s001.zip › Figure S2.pdf]
